# Comparing the effects of even‐ and uneven‐aged silviculture on ecological diversity and processes: A review

**DOI:** 10.1002/ece3.3737

**Published:** 2017-12-20

**Authors:** Philippe Nolet, Daniel Kneeshaw, Christian Messier, Martin Béland

**Affiliations:** ^1^ Institut des Sciences de la Forêt tempérée (ISFORT) Université du Québec en Outaouais Ripon QC Canada; ^2^ Département des Sciences Biologiques Centre d’étude de la Forêt (CEF) Université du Québec à Montréal Montréal QC Canada; ^3^ Département des Sciences Naturelles Institut des Sciences de la Forêt Tempérée (ISFORT) Centre d’étude de la Forêt (CEF) Université du Québec en Outaouais (UQO) Ripon QC Canada; ^4^ École de Foresterie Université de Moncton, Campus d'Edmundston Edmundston NB Canada

**Keywords:** biodiversity, conservation, ecological indicators, ecological processes, even‐aged silviculture, spatial scale, timescale, uneven‐aged silviculture

## Abstract

With an increasing pressure on forested landscapes, conservation areas may fail to maintain biodiversity if they are not supported by the surrounding managed forest matrix. Worldwide, forests are managed by one of two broad approaches—even‐ and uneven‐aged silviculture. In recent decades, there has been rising public pressure against the systematic use of even‐aged silviculture (especially clear‐cutting) because of its perceived negative esthetic and ecological impacts. This led to an increased interest for uneven‐aged silviculture. However, to date, there has been no worldwide ecological comparison of the two approaches, based on multiple indicators. Overall, for the 99 combinations of properties or processes verified (one study may have evaluated more than one property or process), we found nineteen (23) combinations that clearly showed uneven‐aged silviculture improved the evaluated metrics compared to even‐aged silviculture, eleven (16) combinations that showed the opposite, and 60 combinations that were equivocal. Furthermore, many studies were based on a limited study design without either a timescale (44 of the 76) or spatial (54 of the 76) scale consideration. Current views that uneven‐aged silviculture is better suited than even‐aged silviculture for maintaining ecological diversity and processes are not substantiated by our analyses. Our review, by studying a large range of indicators and many different taxonomic groups, also clearly demonstrates that no single approach can be relied on and that both approaches are needed to ensure a greater number of positive impacts. Moreover, the review clearly highlights the importance of maintaining protected areas as some taxonomic groups were found to be negatively affected no matter the management approach used. Finally, our review points to a lack of knowledge for determining the use of even‐ or uneven‐aged silviculture in terms of both their respective proportion in the landscape and their spatial agency.

## 1 INTRODUCTION

Forests are used primarily for harvesting wood to fulfill human needs but they also provide important habitats to two‐thirds of terrestrial organisms (Duraiappah, Naeem, Agardy, & Assessment, 2005), and are thus of conservation concern. As the human population is projected to reach 8.2 billion by 2030, the demand for wood products will also inevitably increase (FAO, 2009), intensifying the pressure on nonprotected forests to be managed for wood production. In such a context, conservation areas may fail to maintain terrestrial biodiversity if they are not supported by the surrounding managed forest matrix. However, the contribution of the managed forest matrix to biodiversity conservation depends on silvicultural practices providing suitable habitats and maintaining ecological processes (Messier, Puettmann, & Coates, 2013).

**Figure 1:**
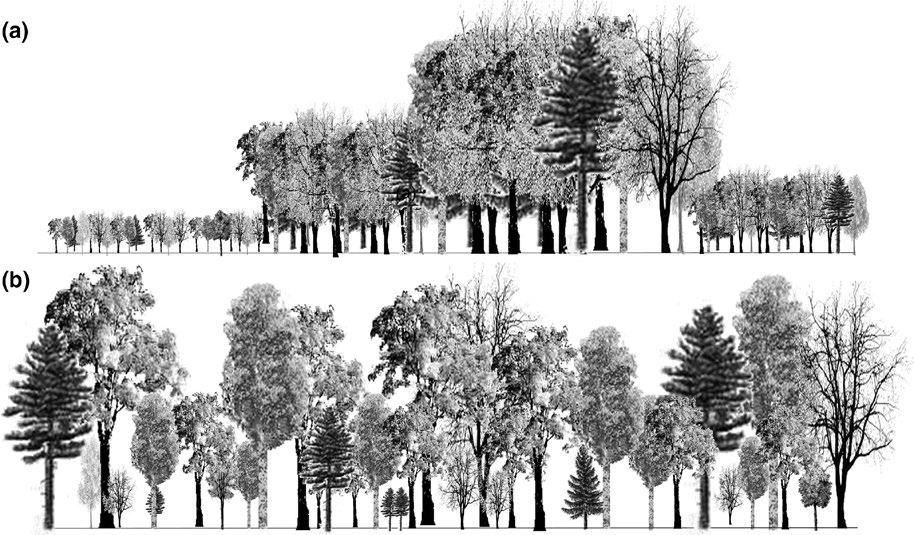
Schematic representation of the difference at the stand scale between (a) stands subjected to even‐aged silviculture at four different developmental stages and (b) stand subjected to uneven‐aged silviculture. Following even‐aged silviculture, trees in each stand are surrounded by trees of a similar age and height, while in uneven‐aged silviculture, trees are of varying ages and heights. In both cases, smaller trees are expected to replace larger trees once the latter are harvested

In recent decades, there has been rising public pressure around the world against the systematic use of even‐aged silviculture—which often implies clear‐cutting—because of its perceived negative esthetic and ecological impacts (Schütz, Pukkala, Donoso, & von Gadow, 2012). It has also been shown that the overuse of even‐aged techniques has led to changes in forest structure and biodiversity compared to natural systems (Bergeron, Leduc, Harvey, & Gauthier, 2002; Cyr, Gauthier, Bergeron, & Carcaillet, 2009; Paillet et al., 2010). Many authors have proposed alternatives to large‐scale industrial forestry operations based on clear‐cutting, ranging from ensuring better protection of key elements within managed ecosystems (Franklin, Berg, Thornburgh, & Tappeiner, 1997; Gustafsson et al., 2012), devoting an increased proportion of landscapes to forest ecosystem conservation (Côté et al., 2010; Seymour & Hunter, 1992), and decreasing the use of even‐aged silviculture in favor of uneven‐aged silviculture (O'Hara, 2002; Schütz et al., 2012) (see Panel 1 and Figure 1 for a brief description of even‐ and uneven‐aged silviculture).

Panel 1 **Brief description of even‐ and uneven‐aged silviculture**

1 Even‐aged silviculture is a set of silvicultural treatments that favor the regrowth of a stand dominated by trees that are mostly of the same age. Uneven‐aged silviculture is a set of silvicultural treatments that favor regrowth of at least three age classes (Helms, 1998). The two approaches differ in their implementation spatially and temporally. Even‐aged management implies a clear‐cut, or a final cut that resets the stand to a regeneration stage. Uneven‐aged management implies repeated partial cuts that regenerate the stand more continuously and leave some permanent forest cover. Because the amount of timber harvested per unit of surface in one entry is not the same for both approaches, for a same amount of timber harvested, the footprint in the forest that is left by the two systems differs (Figure 1).

In Europe, for example, the direct transformation of existing even‐aged plantations to mixed, uneven‐aged managed forests has been seen in recent decades (Pommerening & Murphy, 2004). However, this switch to continuous cover uneven‐aged forestry has led to concerns about the potential reduction or loss of tree species that are shade‐intolerant, such as oak (Ligot, 2014). The reasons to support one system over the other vary; it can be for social reasons (Ehrenhaldt, 1994) or ecological reasons (Seymour, White, & deMaynadier, 2002). As the proportion of natural landscapes decreases worldwide, it will be critical to understand how biodiversity and ecological processes respond to the two dominant silvicultural approaches. A thorough and unbiased comparison of the existing literature on the effects of these two approaches on diversity and key ecological functions is lacking.

Ecologically, many authors continue to make the assumption that biodiversity can be protected by having harvesting operations emulate the natural disturbance regimes and the ensuing natural forest structures (Bergeron, Harvey, Leduc, & Gauthier, 1999; Franklin & Forman, 1987; Hunter, 1993). This hypothesis has led to a movement to emulate natural conditions through forest management (Gauthier et al., 2009), and emulating natural disturbances generally implies the use of a diverse silviculture (Kuuluvainen, 2002). However, although there are theoretical and partially evidenced pathways to support the use of a diverse silviculture to maintain biodiversity and ecological processes (Franklin & Forman, 1987), these pathways are far from confirmed.

Most studies to date comparing even‐ and uneven‐aged silviculture have focused on the response of a small group of species. There is a need, however, to move from an understanding of the responses of individual entities or groups of taxa or processes to a more holistic evaluation. What is the evidence that even‐ or uneven‐aged silviculture facilitates or impedes particular taxa or processes? Does one system consistently maintain biodiversity or some taxa and processes? Or alternatively, do both systems modify forests so that biodiversity, or at least some taxa, and ecological processes will not be protected outside of conservation areas? To answer these questions, we first present a literature review of scientific papers that include a comparison between effects of even‐ and uneven‐aged silviculture on major taxonomic groups and several ecological processes, covering various types of forests from different forest biomes around the world. Secondly, we discuss key insights provided by the review that can be used to better plan and manage interventions.

## 2 LITERATURE REVIEW

### 2.1 Approach and rationale

**Figure 2:**
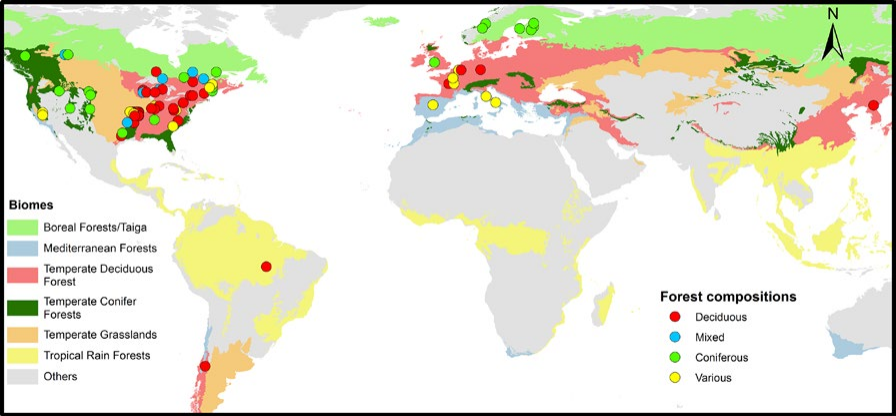
Approximate location and species composition of the reviewed studies in relation to forest biomes. The term “Various” means that studies were conducted in more than one forest composition

To perform the literature review, we searched for scientific papers (rather than dissertations or technical reports, which are not always in scientific databases) that included a comparison between the effects of even‐ and uneven‐aged silviculture on one or many ecological indicators (Elbakidze, Angelstam, Andersson, Nordberg, & Pautov, 2011; Kneeshaw et al., 2000). We used comparisons from a diversity of forest ecosystems worldwide, where such comparisons were available, to provide the most complete overview of the information readily (i.e., in English and indexed on most popular databases) available to scientists and practitioners for comparing even‐ and uneven‐aged silviculture. This initial search was complemented by meta‐analyses and reviews that dealt with the impact of canopy removal intensity on ecological indicators. Our search was performed using three different scientific databases: Web of Science, Scopus, and Google Scholar. Because many words other than “even‐aged” and “uneven‐aged silviculture” may be used to refer to these systems (e.g., clear‐cut, selection cut), and because keywords did not always indicate a comparison between the two silvicultural approaches, an “automated” search could not be used. Instead, several hundred abstracts that contained the terms “even‐aged,” “uneven‐aged,” “selection cut,” or “clear‐cut” were carefully examined to determine whether there was an even‐ and uneven‐aged ecological comparison. Some studies might have been unintentionally omitted from this review as forest ecologists do not always use forestry terms to define the silvicultural treatment under study. A sample of the results we were able to obtain from the review is provided in Table 1, while detailed results of the complete review are provided as Table S1. For each paper, the main metrics used to compare the silvicultural approaches were listed and whether or not a system (EAS or UAS) “improved” the metrics (compared to the other one) was evaluated. If the results were variable across metrics or across species, the results were reported as equivocal. However, when summarizing a paper, we attempted to remain true to the interpretations of results as reported by the authors regarding the compared effects of even‐ and uneven‐aged silviculture. Moreover, for each paper, we evaluated how time and spatial scales were considered: Whether the study design was based on a one‐time or a multi‐temporal assessment; and whether or not it was specifically noted that results at the stand scale could be translated to the landscape scale automatically. Despite our goal of being geographically representative, of the 76 studies reviewed, most are concentrated in the Northern Hemisphere and mostly in the Americas. Although the studies were conducted in forests of varying species composition, most were conducted in forests that were dominated by deciduous tree species (Figure 2).

**Table 1:** Sample of the literature analysis comparing even‐ (EAS) and uneven‐aged silviculture (UAS) for various ecosystem properties and processes

Ecosystem component	Properties or processes studied	Metrics used	System improving the evaluated metrics	Spatial scale	Timescale	Reference
Tree species	Saplings	Shannon index	EAS	Stand only	No	Niese and Strong (1992)
Herbs and shrubs	Spring and summer herbs	Various	Equivocal	Stand only	No	Kern et al. (2006)
Structure	Down woody debris, dead standing trees, and snags	Density	UAS	Stand only	No	Atlegrim and Sjöberg (2004)
Bryophytes	Ectomycorrhizal fungi	Richness	UAS	Stand only	No	Kropp and Albee (1996)
Mammals	Small mammals population	Abundance, reproduction, and survival	Equivocal	Stand only	No	Klenner and Sullivan (2009)
Birds	Early‐succession species	Abundance	EAS	Stand only	Yes	Perry and Thill (2013)
Herpetofauna	Red‐backed salamander	Abundance	UAS	Stand only	No	Hocking et al. (2013)
Invertebrates	Dung beetle	Various	Equivocal	Stand+Landscape	No	Masís and Marquis (2009)
Carbon	Carbon sequestration	Total ecosystem amount of carbon	Equivocal	Stand+Landscape	Yes	Pukkala et al. (2011)
Soil	Soil properties	Soil density, pH, C, N, Ca, Mg, K, CEC	Equivocal	Stand only	No	Elliott and Knoepp (2005)

The complete table is presented in Table S1.

### 2.2 Analysis by ecosystem component

A surprisingly limited number of studies were identified that compared the effects of even‐ and uneven‐aged silviculture on tree species diversity and composition (Table 2). Of the eight studies found, only one (Table 2) showed that UAS was preferable (based on the metrics evaluated) to EAS (Torras & Saura, 2008), while a few of the studies revealed that EAS favored tree species diversity (Doyon, Gagnon, & Giroux, 2005; Messina et al., 1997). Niese and Strong (1992) even considered that uneven‐aged silviculture may lead to dominance or monocultures of late‐successional species. A high species richness can be considered positive for forest resilience as it contributes to spread the risk in case of a major stress or perturbation (Millar, Stephenson, & Stephens, 2007) as long as the new species composition continues to provide important ecosystem services (i.e., Holling's reorganization phase; Drever, Peterson, Messier, Bergeron, & Flannigan, 2006). However, in terms of maintaining some key functional traits, the loss of species is a concern if the rotations that are used for even‐aged silviculture are too short for the recruitment of late‐successional species. Even‐aged silviculture can then lead to major shifts at the landscape or regional scale if the matrix shifts from one that is dominated by late‐successional to early‐successional species (Gauthier et al., 2009).

**Table 2:** Summary of the studies comparing EAS and UAS

Ecosystem component	System improving the evaluated metrics		
	EAS	UAS	Equivocal
Birds	4	1	9
Bryophytes and others	1	3	3
Carbon	2	6	4
Herbs and shrubs	2		11
Herpetofauna		3	6
Invertebrates	2	2	11
Mammals	2		5
Soil		3	3
Structural elements		4	4
Tree species	3	1	4
Total	16	23	60

Understory (shrubs and herbs) species diversity has been studied much more than tree species diversity with regard to even‐ and uneven‐aged silviculture. From the literature review, none of the studies (Table 2) clearly states that UAS was preferable to EAS in terms of understory species diversity and composition. A few studies have, however, reported that uneven‐aged silviculture could trigger the development of a dense shrub layer (Decocq et al., 2004), a phenomenon observed worldwide that strongly influences understory forest dynamics (Royo & Carson, 2006). Even‐aged silviculture, on the other hand, may have a strong impact on understory plant species composition in the short term (Haeussler, Bergeron, Brais, & Harvey, 2007). This short‐term impact may be influenced by the level of soil disturbance incurred during harvesting operations (Kern, Palik, & Strong, 2006). Over the longer term and using a meta‐analysis, Duguid and Ashton (2013) showed that the effect of even‐aged silviculture on plant richness depended upon the developmental stage, with the lowest diversity found at the understory reinitiation stage (about 30–50 years after harvest). These results highlight the importance of studying various stand developmental stages after even‐aged silviculture when making comparisons with uneven‐aged silviculture as differences between the two systems may vary, depending on the even‐aged developmental stage (Figure 1) to which the comparison is made.

For structural elements, four of the eight studies showed that UAS was preferable to EAS (Table 2). However, it appeared that results depended upon the ecosystem being studied, the manner in which treatments were implemented, and the manner in which data were collected (e.g., minimum diameter of down woody debris). Yet it is clear from this review that an overall loss of structural diversity occurs in managed forests for both even‐ and uneven‐aged systems. It appears that neither approach (as applied in these studies) was able to maintain the structural diversity found in natural forests.

With only seven studies comparing mycorrhizae, lichens, bryophytes, fungi and bacterial communities (Table 2), it is impossible to draw a clear conclusion on the effects of even‐ and uneven‐aged silviculture on these ecosystem components. These elements of the forest ecosystem definitely require deeper attention, especially mycorrhizae because of their fundamental importance to the functioning of forest ecosystems (Simard, 2009).

Both for mammal and bird populations, only one of the 21 studies (Table 2) clearly showed that UAS was preferable to EAS; responses to both approaches appeared species‐specific and not generalizable across all taxa indicating no consistent pervasive effect of either management type. Nonetheless, birds (Morris, Porneluzi, Haslerig, Clawson, & Faaborg, 2013) are more strongly associated with distinct forest development stages and forest structures than are mammals. It also appeared that uneven‐aged silviculture that is practiced uniformly across a landscape reduces avian diversity (Thill & Koerth, 2005), while this trend is not as clear for mammals. However, the number of studies that compared (even‐ vs. uneven‐aged silviculture) birds and mammals at the landscape level is rare (but see Becker et al., 2011). As dispersion processes are important for these taxa, there is a clear need to compare landscapes that are mainly managed through even‐aged silviculture to others mainly managed through uneven‐aged silviculture and to do so at a scale corresponding to the taxa's respective home ranges. For both taxonomic groups, time since treatment is also important. For example, Thornton, Wirsing, Roth, and Murray (2012), in coniferous forests in Idaho, showed that in the short term, both clear‐cuts and partial cuts negatively affected snowshoe hare (*Lepus americanus*). However, older clear‐cuts (15–40 years old) were the best habitat for this species. In oak forests of Missouri, Morris et al. (2013) observed that some early negative effects of both even‐ and uneven‐aged silviculture on some bird populations were still apparent 14 years after harvest.

**Figure 3:**
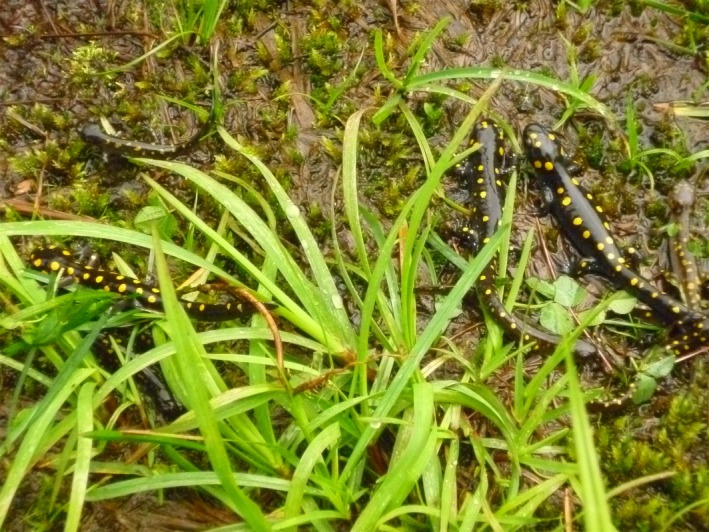
Herpetofaunal communities, represented here by spotted salamanders (*Ambystoma maculatum*), are often affected by both even‐ and uneven‐aged silviculture. Photograph: Marie‐Ève Roy

For herpetofaunal communities (Figure 3), any kind of forest management, that is, uneven‐aged or even‐aged, appeared detrimental in many cases (Hocking et al., 2013; Homyack & Haas, 2009) (Table 2). In fact, positive responses of herpetofaunal species to any kind of logging are rarely observed, especially toward clear‐cuts at the stand scale. Tilghman, Ramee, and Marsh (2012) observed that populations generally recovered as the forest regenerated, while Homyack and Haas (2009) observed no recovery 13 years after harvesting following the either even‐ or uneven‐aged silviculture. Due to the more frequent number of harvest entries in uneven‐aged selection cutting systems compared to even‐aged silviculture, these authors were doubtful of any environmental benefits following selection cuts at the landscape level (Homyack & Haas, 2009). This, as for structural elements, highlights the importance of maintaining unmanaged forests in the landscape for this group of species.

According to our review, invertebrates do not show a consistent response to the silvicultural system being used (Table 2). However, populations appear to be sensitive to forest management in general (Summerville, 2011; de Warnaffe & Lebrun, 2004). For example, the study of Latty, Werner, Mladenoff, Raffa, and Sickley (2006) in Michigan and Wisconsin showed that beetle communities in stands that were recently managed using uneven‐aged silviculture were very different from those managed using even‐aged silviculture and from those of old‐growth forests, even if there were few species that were strictly associated with the different types of disturbance history. The authors estimated that at the landscape scale, insect species that preferred old‐growth forests have declined to a large extent.

For carbon‐related processes, six of the twelve studies (Table 2) identified uneven‐aged silviculture preferable to even‐aged silviculture. However, two of the studies examined the total amount of carbon in the ecosystem very shortly after harvesting (Lee, Morrison, Leblanc, Dumas, & Cameron, 2002) instead of considering it over a full rotation (Nilsen & Strand, 2013). The most complete studies, based upon simulations (Moore, DeRose, Long, & van Miegroet, 2012; Nunery & Keeton, 2010; Pukkala, Lähde, & Laiho, 2011) or long‐term measurements (Nilsen & Strand, 2013), provide equivocal results in terms of the best silvicultural approach to sequester or store carbon. These contradictions may be due to the complexity of the calculations, as acknowledged by Moore et al. (2012), who emphasized that the accuracy of their results depended upon several factors, including the forest products that were generated. Nunery and Keeton (2010) even showed that the comparison between even‐ and uneven‐aged silviculture is influenced by the level of structural retention applied in the treatments and also differs if carbon storage is considered instead of carbon sequestration.

Three of the six studies (Table 2) showed that uneven‐aged silviculture is preferable to even‐aged silviculture for soil processes and functions, and the effects appear twofold. On the one hand, the effects of both silvicultural approaches on soil chemistry or density are limited (e.g., Elliott & Knoepp, 2005). On the second hand, when examining stream water, strong effects are observed. Siemion, Burns, Murdoch, and Germain (2011), in New York State, showed that above a certain harvesting intensity (about 40% of the watershed), nitrate and calcium concentrations in stream water increased linearly with harvesting intensity. Above this threshold—most likely to be encountered in even‐aged silviculture—concentrations increased more rapidly than harvesting intensity. Wang, Burns, Yanai, Briggs, and Germain (2006), also in New York, observed a roughly linear relationship between harvesting intensity and changes in aluminum, calcium, and magnesium concentrations in stream water. Changes in concentrations of nitrate (about 5×) and potassium (about 100×) were not linear but increased exponentially with harvesting intensity. Stream water chemistry returned to near preharvest conditions about 1 year after harvest, except for nitrate concentrations. Although they occur for only a very limited time, these increased levels of nutrients are liable to shift ecosystem states (watercourses in this case; e.g., Rask, Nyberg, Markkanen, & Ojala, 1998).

Overall, for the 99 combinations of properties or processes verified (one study may have evaluated more than one property or process), we found 23 combinations that clearly showed uneven‐aged silviculture improved the metrics compared to even‐aged silviculture, sixteen (16) combinations that showed the opposite, and 60 combinations that were equivocal. We acknowledge that there is some subjectivity in such an analysis as a few studies might have been analyzed differently by other evaluators. Furthermore, it is important to note that many studies were based on a limited study design without either a timescale (44 of the 76) or spatial (54 of the 76) scale consideration (Table 3). Yet, we are confident in the general portrait that we present.

**Table 3:** Number of reviewed studies that considered timescale and spatial scale

Timescale consideration	Spatial scale consideration		Total
	Stand only	Stand + landscape	
No	39	5	44
Yes	15	17	32
Total	54	22	76

## 3 DISCUSSION

This systematic review of the literature is the first to compare even‐ and uneven‐aged silviculture across ecosystems using multiple ecological indicators. Based on the literature review, we conclude that the commonly held view (Stout, 1998), as well as the shift in practices in Europe (Pommerening & Murphy, 2004), suggesting that uneven‐aged silviculture is better suited than even‐aged silviculture to maintaining ecological diversity and processes is not substantiated by our analyses. This conclusion is in accordance with the multitaxa even‐ versus uneven‐aged comparison performed by Schall et al. (2018).

Instead, our analyses reveal both strengths and weaknesses, in terms of ecological impacts, for both even‐aged and uneven‐aged silviculture. They show that the impacts are both scale‐ and organism‐dependent. For example, even‐aged silviculture better promotes tree and plant species (alpha) diversity, as well as maintains more shade‐intolerant trees, at the stand scale than uneven‐aged silviculture. On the other hand, clear‐cutting/even‐aged silviculture appears to reduce the number of mycorrhizal fungi (Kropp & Albee, 1996), lichens, and bryophytes (Paillet et al., 2010) and to affect soil integrity (Spinelli, Magagnotti, & Nati, 2010) and surface water runoff (Wang et al., 2006). Our review shows that responses to even‐ and uneven‐aged silviculture can also be species‐specific within a taxon. A key finding is that many studies analyzed in this article showed that both even‐ and uneven‐aged silviculture have negative impacts on some species, for example, herpetofaunal species, and processes when compared to unmanaged stands. This may mean that no matter the silvicultural approach used, some species will be negatively affected. Alternatively, it may also mean that when the focus is on the even‐ and uneven‐aged comparison, the most important factor for these species/processes, that is, soil disturbance, human presence, may be missed.

### 3.1 The complexity of comparing even‐ and uneven‐aged silviculture

The greatest challenge when comparing even‐ and uneven‐aged silviculture is the consideration of spatial scale and temporal scale (Kuuluvainen, Tahvonen, & Aakala, 2012). To harvest the same volume of wood, the area affected by uneven‐aged silviculture is much larger (e.g., 3–5 times, assuming similar productivity between the two systems) than the area affected by even‐aged silviculture. This was rarely considered in the papers we analyzed. Furthermore, scaling up from the stand to the landscape scale is often much more complex than a simple multiplication. The spatial assemblage of forest stands is especially important for taxonomic groups such as birds (Becker et al., 2011) and vertebrates in general (Tews et al., 2004). Nevertheless, evaluating the cumulative effects of implementing either system over a whole landscape is a difficult task, and for economic and logistical reasons has not yet been undertaken.

Moreover, although stand characteristics and composition change\recover over time (i.e., stand development), most comparisons were drawn at only one moment in time (Table 3). A full comparison would require evaluating effects of even‐aged and uneven‐aged harvesting over one full even‐aged stand rotation (say, 80–100 years) and for an equivalent time for uneven‐aged stands over many cutting cycles (3–5 cycles) (Nolet & Béland, 2017).

Furthermore, both approaches encompass various silvicultural subsystems. Uneven‐aged silviculture includes many forms of selection silviculture from gap and group to single‐tree selection cutting, and with variable removal intensities and return intervals. Even‐aged silviculture comprises clear‐cut, seed tree, and shelterwood systems, and it is more and more implemented with some form of retention (Lindenmayer et al., 2012). Moreover, many commercial thinnings (partial cuts) may be implemented during a rotation in even‐aged silviculture. Hence, the ecological effects observed may be quite different among the subsystems.

Overall, the complexity of comparing even‐ and uneven‐aged silviculture may explain the surprisingly limited number of studies that compare ecological effects of even‐ and uneven‐aged silviculture.

### 3.2 Insights for forest management

The bulk of scientific papers comparing UAS to EAS supports arguments (Doyon et al., 2005; Duguid & Ashton, 2013; Gauthier et al., 2009; Hunter, 1993) for using a variety of silvicultural approaches at the landscape level. Although this has been argued based on intuition or specific local studies, our review, based on a large range of indicators and many different taxonomic groups, clearly demonstrates that no single approach can be relied on and that both approaches are needed to ensure a greater number of positive impacts. Our review also clearly shows the importance of maintaining protected areas as some taxonomic groups were found to be negatively affected no matter the form of management used. In the intervening matrix, a variety of silvicultural approaches will provide a gradient of conditions for less sensitive species.

Even‐ and uneven‐aged silviculture, by creating contrasting environmental conditions, will also permit us to better understand species autecology and ecological processes. In turn, this understanding may allow forest ecologists and practitioners to plan spatial arrangements (or other variations in implementation) of even‐ and uneven‐aged silviculture that can maintain species and processes that might otherwise be neglected by the use of a single approach. A simple example is the successful implementation of small clear‐cuts within uneven‐aged managed stands (gap selection, Raymond, Munson, Ruel, & Nolet, 2003; Webster & Lorimer, 2005) to favor mid‐tolerant species regeneration. Such spatial arrangement can be envisioned for other taxa/processes. Hence, we not only propose the use of both even‐ and uneven‐aged silviculture, but also different spatial arrangements in combination with other factors that can positively affect species and ecological processes (e.g., regeneration mode, soil/habitat protection, variable retention).

### 3.3 Future research

Factors that cause decline in some species following any type of forest management should be further studied as it may be possible to mitigate some of these effects. For example, where soil disturbance is a factor, comparing winter versus summer harvest by silvicultural system could help mitigate some negative effects. As vernal pools (and their surroundings) are critical for herpetofaunal communities (a group responding negatively to all silvicultural approaches), it could be worthwhile to implement study designs where the interaction between harvesting and the pools is explicitly evaluated (e.g., even‐ vs. uneven‐aged silviculture and vernal pools protected vs. unprotected). Such designs would help to disentangle the effects of multiple factors and contribute to the development of silvicultural guidelines to protect more fragile species outside conservation areas.

This idea of disentangling confounding effects applies to other situations as well. For example, in most studies that were reviewed, even‐aged silviculture was not followed by tree planting. As tree planting generally implies site preparation and some level of control of competing vegetation (Buitrago, Paquette, Thiffault, Bélanger, & Messier, 2015), it is likely that it has a stronger ecological effect than does natural regeneration‐based even‐aged silviculture. There is clearly a need to discriminate between the effects due to the approach used (even‐ vs. uneven‐aged) and the effects of the regeneration mode and site preparation used. In this way, several authors (e.g., Kern et al., 2006) observed limited differences between even‐and uneven‐aged silviculture in herb species communities and wondered whether soil disturbance was the most important factor. In such situations, studies comparing even‐ versus uneven‐aged silviculture with contrasting soil protection (e.g., with or without snow cover) could better inform management practices.

Finally, large‐scale temporal and spatial comparisons that include an implicit evaluation of the various stand developmental stages created by even‐aged silviculture at the landscape scale (gamma diversity) will be key to understanding the impact on species with needs for multiple habitat types or for species with large home ranges.

## DATA ACCESSIBILITY

Data have not been archived because this article does not contain data.

## AUTHORS’ CONTRIBUTION

PN performed the literature review and produced all the table and figures. PN and DK led the writing of the manuscript. All authors contributed critically to the drafts and gave final approval for publication.

## CONFLICT OF INTEREST

None declared.
